# Structure of nitrilotriacetate monooxygenase component B from *Mycobacterium thermoresistibile*

**DOI:** 10.1107/S1744309111012541

**Published:** 2011-08-16

**Authors:** Y. Zhang, T. E. Edwards, D. W. Begley, A. Abramov, K. B. Thompkins, M. Ferrell, W. J. Guo, I. Phan, C. Olsen, A. Napuli, B. Sankaran, R. Stacy, W. C. Van Voorhis, L. J. Stewart, P. J. Myler

**Affiliations:** a Seattle Structural Genomics Centre for Infectious Disease (SSGCID), USA; b Seattle Biomedical Research Institute, 307 Westlake Avenue North, Suite 500, Seattle, WA 98109, USA; c Emerald BioStructures Inc., 7869 NE Day Road West, Bainbridge Island, WA 98110, USA; d School of Medicine, University of Washington, Seattle, WA 98195, USA; e Berkeley Center For Structural Biology, Ernest Orlando Lawrence Berkeley National Laboratory, 1 Cyclotron Road, Berkeley, CA 94720, USA

**Keywords:** SSGCID, *Mycobacterium thermoresistible*, nitrilotriacetate monooxygenase component B, flavin reductases, oxidoreductases

## Abstract

*Mycobacterium tuberculosis* belongs to a large family of soil bacteria which can degrade a remarkably broad range of organic compounds and utilize them as carbon, nitrogen and energy sources. It has been proposed that a variety of mycobacteria can subsist on alternative carbon sources during latency within an infected human host, with the help of enzymes such as nitrilotriacetate monooxygenase (NTA-Mo). NTA-Mo is a member of a class of enzymes which consist of two components: A and B. While component A has monooxygenase activity and is responsible for the oxidation of the substrate, component B consumes cofactor to generate reduced flavin mononucleotide, which is required for component A activity. NTA-MoB from *M. thermoresistibile*, a rare but infectious close relative of *M. tuberculosis* which can thrive at elevated temperatures, has been expressed, purified and crystallized. The 1.6 Å resolution crystal structure of component B of NTA-Mo presented here is one of the first crystal structures determined from the organism *M. thermo­resistibile*. The NTA-MoB crystal structure reveals a homodimer with the characteristic split-barrel motif typical of flavin reductases. Surprisingly, NTA-MoB from *M. thermoresistibile* contains a C-terminal tail that is highly conserved among myco­bacterial orthologs and resides in the active site of the other protomer. Based on the structure, the C-terminal tail may modulate NTA-MoB activity in mycobacteria by blocking the binding of flavins and NADH.

## Abstract 2

The 1.6 Å resolution crystal structure of nitrilotriacetate monooxygenase component B (NTA-MoB) from *M. thermoresistibile* is presented, revealing a highly conserved C-terminal tail that may modulate the activity of NTA-MoB in mycobacteria.

## 1. Introduction

Bacteria within the *Mycobacterium* genus include *M. tuberculosis*, the pathogen responsible for tuberculosis (TB), a disease which has infected millions worldwide (Anderton *et al.*, 2006 ▶; Rylance *et al.*, 2010 ▶). This highly contagious disease is responsible for three million deaths per year and highly regulated facilities are needed to study it owing to its ease of transmission. Over 120 species of *Mycobacterium* have been identified to date, many of which can cause disease, particularly in individuals with suppressed or compromised immunity (Neonakis *et al.*, 2007 ▶). *M. thermoresistibile* is a non-tuberculous species of *Mycobacterium* which has had multiple pathology reports over the years, including a recent identification in an infected patient (Neonakis *et al.*, 2009 ▶). As its name implies, *M. thermoresistibile* thrives at elevated temperatures and unlike most other mycobacteria it can survive in culture at 333 K for up to 4 h (Weitzman *et al.*, 1981 ▶; Tsukamura, 1966 ▶). Although relatively rare, increasing evidence of its ability to infect humans and the fact that it shares many homologous genes with *M. tuberculosis* warrants further study of this pathogenic organism for possible therapeutic intervention (Weitzman *et al.*, 1981 ▶; Kremer *et al.*, 2002 ▶; Boloorsaz *et al.*, 2006 ▶).

Mycobacteria are named for their ability to produce mycolic acid, and bacteria from this genus are capable of degrading a wide range of organic compounds (Savvi *et al.*, 2008 ▶). Like other microorganisms, various mycobacteria can use small organic compounds such as nitrilotriacetate (NTA) as their sole source of nitrogen, carbon and energy, allowing rapid adaptation to varying conditions within the host (Uetz *et al.*, 1992 ▶; Bally *et al.*, 1994 ▶). Recent reports predicted that *M. tuberculosis* could subsist on alternative carbon sources during persistence within the human host, specifically during its macrophage infection period, where by nature it needs to endure glucose deficiency and an abundance of fatty acids. The complex repertoire of genes involved in lipid metabolism in *Mycobacterium* is thus a key factor in its strong pathogenicity (Van der Geize *et al.*, 2007 ▶; Savvi *et al.*, 2008 ▶). Nitrilotriacetate monooxygenase (NTA-Mo) is an oxidoreductase and a member of the family of two-component monooxygenases which initiates the oxidation of NTA under aerobic conditions (van Berkel *et al.*, 2006 ▶). This enzyme is comprised of two parts: component A (NTA-MoA), which has monooxygenase activity and is responsible for the oxidative conversion of NTA to iminodiacetate (IDA) and glyoxylate, and component B (NTA-MoB), a flavin reductase which consumes NADH to reduce FMN to FMNH_2_, which is a required cofactor in the oxidization step. The combined NTA-Mo assembly as whole is categorized as a class C flavoprotein monooxygenase (van Berkel *et al.*, 2006 ▶). The amino-acid alignment and the three-dimensional structure motif of NTA-MoB from *M. thermoresistibile* (*Mth*NTA-MoB) associate it with a family of short-chain flavin reductases. This group of proteins exists in many eukaryotic and prokaryotic organisms, including all mycobacteria (Knobel *et al.*, 1996 ▶). Here, we report the 1.6 Å resolution crystal structure of *Mth*NTA-MoB, a homolog of Rv3567c from *M. tuberculosis* (*Mtu*) in a highly conserved family within *Mycobacterium*. At the time of writing, it is one of only five entries for *M. thermoresistibile* available in the Protein Data Bank (PDB), all of which have been solved by the Seattle Structural Genomics Center for Infectious Disease (SSGCID).

## 2. Methods

### 2.1. Protein expression and purification

The gene for the full-length NTA-MoB protein (Target DB MythA.00250.a; GenBank accession No. HQ644138; NCBI YP_890259.1; A0R521 homolog) spanning residues 1–189 (‘ORF’) was amplified from *M. thermoresistibile* Tsukamura strain ATCC19527/NCTC10409 (genomic DNA and sequence information provided by Dr Christoph Grundner, Seattle Biomedical Research Institute) and cloned into a pAVA0421 vector encoding an N-terminal hexahistidine-affinity tag followed by the human rhinovirus 3C protease cleavage sequence (MAHHHHHHMGTLEAQTQGPGS-ORF; Alexandrov *et al.*, 2004 ▶) by ligation-independent cloning (LIC; Aslanidis & de Jong, 1990 ▶). The plasmid construct for *Mth*NTA-MoB (MythA.00250.a.A1) was transformed into *Escherichia coli* BL21 (DE3) Rosetta cells. An overnight culture was grown in LB broth at 310 K and was used to inoculate 2 l ZYP-5052 auto-induction medium, which was prepared as described by Studier (2005 ▶). *Mth*NTA-MoB protein was expressed in a LEX bioreactor in the presence of antibiotics. After 24 h at 298 K, the temperature was reduced to 288 K for a further 60 h. The sample was centrifuged at 4000*g* for 20 min at 277 K and the cell paste was flash-frozen in liquid nitrogen and stored at 193 K.

For purification, the frozen cell pellet was thawed and completely resuspended in lysis buffer (20 m*M* HEPES pH 7.4, 300 m*M* NaCl, 5% glycerol, 30 m*M* imidazole, 0.5% CHAPS, 10 m*M* MgCl_2_, 3 m*M* β-­mercaptoethanol, 1.3 mg ml^−1^ protease-inhibitor cocktail, 0.05 mg ml^−1^ lysozyme). The resuspended cell pellet was then disrupted on ice for 15 min with a Branson Digital 450D Sonifier (70% amplitude, with alternating cycles of 5 s pulse-on and 10 s pulse-off). The cell debris was incubated with 20 µl Benzonase nuclease at room temperature for 40 min. The lysate was clarified by centrifugation at 277 K with a Sorvall RC5 at 10 000 rev min^−1^ for 60 min. The clarified solution was filtered through a 0.45 µm syringe filter (Corning Life Sciences, Lowell, Massachusetts, USA). The lysate was purified by IMAC using a HisTrap FF 5 ml column (GE Biosciences, Piscataway, New Jersey, USA) equilibrated with binding buffer (25 m*M* HEPES pH 7.0, 300 m*M* NaCl, 5% glycerol, 30 m*M* imidazole, 1 m*M* TCEP) and eluted with 500 m*M* imidazole in the same buffer. *Mth*NTA-MoB was concentrated without 3C protease cleavage of the hexahistidine tag. The concentrated pool was further resolved by size-exclusion chromatography (SEC) using a Superdex 75 26/60 column (GE Biosciences) equilibrated with SEC buffer (20 m*M* HEPES pH 7.0, 300 m*M* NaCl, 5% glycerol, 1 m*M* TCEP) attached to an ÄKTA FPLC system (GE Biosciences). Peak fractions were collected and pooled based on purity-profile assessment by SDS–PAGE. Concentrated pure protein in SEC buffer was flash-frozen in liquid nitrogen and stored at 193 K. The final concentration (68.9 mg ml^−1^) was determined by UV spectrophotometry at 280 nm and the final purity (>97%) was assayed by SDS–PAGE.

### 2.2. Crystallization

Crystallization trials were set up according to a rational crystallization approach (Newman *et al.*, 2005 ▶) using the JCSG+ and PACT sparse-matrix screens from Emerald BioSystems and Molecular Dimensions, respectively. 0.4 µl protein solution (68.9 mg ml^−1^) was with mixed with an equal volume of precipitant and set up against 80 µl reservoir solution in sitting-drop vapor-diffusion format in 96-­well Compact Jr plates from Emerald BioSystems at 289 K. Crystals grew in several conditions within 9 d, but the crystal used for data collection grew in the presence of 0.2 *M* magnesium chloride, 0.1 *M* MES pH 6.0 and 20% PEG 6000 (PACT condition B10).

### 2.3. Data collection and structure determination

A crystal was harvested, cryoprotected using precipitant solution supplemented with 20% glycerol and vitrified in liquid nitrogen. A 1.6 Å resolution data set was collected under a stream of liquid nitrogen on Advanced Light Source (ALS) beamline 5.0.2 as part of the ALS Collaborative Crystallography program (Table 1 ▶). The data were reduced with *HKL*-2000 (Otwinowski & Minor, 1997 ▶). The structure (Table 2 ▶) was solved by molecular replacement using *Bacillus thermoglucosidasius* A7 flavin reductase A2 protein-only dimer from molecules *A* and *B* of PDB entry 1rz0 (van den Heuvel *et al.*, 2004 ▶) as a search model in *Phaser* (McCoy *et al.*, 2007 ▶) from the *CCP*4 suite (Winn *et al.*, 2011 ▶). The asymmetric unit was comprised of two independent dimers. The final model was obtained after numerous iterative rounds of refinement in *REFMAC* (Murshudov *et al.*, 2011 ▶) and manual rebuilding in *Coot* (Emsley & Cowtan, 2004 ▶). The final model contained residues Ala3–Ala181 with no internal gaps for protomer *A* and a few additional protein residues for each of the other three protomers. In addition, the final model contained one glycerol molecule (bound to protomer *D*) and 548 water molecules. The structure was assessed and corrected for geometry and fitness using *MolProbity* (Chen *et al.*, 2010 ▶). Data-collection results and structure-refinement statistics are listed in Tables 1 ▶ and 2 ▶.

## 3. Results

### 3.1. Identification of *Mth*NTA-MoB

*Rv3567c* (UniProt accession No. P96849) is a member of a large group of genes that are under the control of a ketosteroid regulon, *kstR*, which is a member of the tetracycline resistant-like family of transcriptional regulators (Kendall *et al.*, 2007 ▶). *Rv3567c* is predicted to be involved in lipid catabolism; this gene has been shown to be inducible both in palmitic acid and when grown on cholesterol in *Rhodococcus*. sp strain RHA1, a soil bacterium related to *M. tuberculosis* (Van der Geize *et al.*, 2007 ▶). *Rv3567c* clusters with about 28 genes specifically expressed in *M. tuberculosis*, among which *Rv3569c* (*hsaD*) has been shown to be essential for survival in primary murine macrophages by a transposon-site hybridization (TraSH) experiment in *M. tuberculosis* H37Rv. For these reasons, *Rv3567c* and related genes have been assigned to the cholesterol-degradation pathway (Van der Geize *et al.*, 2007 ▶; Rengarajan *et al.*, 2005 ▶). Although NTA-MoB tends to be highly conserved across *Mycobacterium* species, only one homolog was found in the UniProt database at the outset of this work: that from *M. smegmatis* (A0R521). A search for NTA-MoB homologs in *M. tuberculosis* in the TubercuList database (http://genolist.pasteur.fr/TubercuList/) resulted in a match to Rv3567c (Cole *et al.*, 1998 ▶). The *Mth*NTA-MoB protein consists of 189 amino-acid residues and is 82% identical (89% similar) to *Mtu*NTA-MoB, which in turn matches 100% to Rv3567c in TubercuList. In addition, *Mth*NTA-MoB is at least 80% identical to all other known *Mycobacterium* orthologs, including that from *M. smegmatis* (A0R521). During target selection, 21 additional paralogs of NTA-MoB were selected and have entered the SSGCID pipeline, including those from *M. abscessus*, *M. avium*, *M. bovis*, *M. leprae*, *M. marinum*, *M. paratuberculosis*, *M. smegmatis* and *M. ulcerans*. Among these 21 homologs, 12 were successfully cloned, expressed and purified and three formed crystals, one of which (*Mth*NTA-MoB) diffracted to sufficiently high resolution for structure determination.

### 3.2. Comparison with other short-chain flavin reductases

A search of the Protein Data Bank (http://www.rcsb.org/pdb/) resulted in no proteins with a sequence similarity greater than 35% to *Mth*NTA-MoB. The Pfam database (http://www.sanger.ac.uk/software/pfam) assigned *Mth*NTA-MoB to the PF01613 family of proteins with the FMN-binding split-barrel motif at an *E* value of 8.7 × 10^−40^. This NADH:FMN oxidoreductase or flavin reductase family was first described in the early 1990s and exists in many organisms, primarily Gram-negative bacteria (Uetz *et al.*, 1992 ▶; Blanc *et al.*, 1995 ▶). These short-chain flavin reductases are involved in a variety of biological reactions and often act in concert with a flavin-dependent monooxygenase which oxidizes through the addition of molecular oxygen (Kirchner *et al.*, 2003 ▶; Galán *et al.*, 2000 ▶). However, they do not share sequence homology with flavin reductases found in *E. coli* or luminous bacteria or with the LuxG protein class found in *lux* operons (Nijvipakul *et al.*, 2008 ▶).

**Figure 1:**
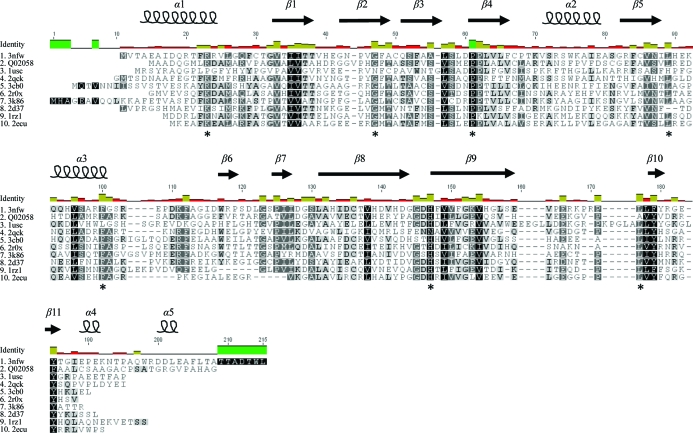
Protein sequence alignment of *Mth*NTA-MoB (PDB entry 3nfw) with other short-chain flavin reductases. The secondary-structural elements of 3nfw are indicated and labelled above the aligned sequences. The similarity levels for each of the amino-acid positions are indicated using a grayscale, where darker shades indicate a higher degree of conservation. Strictly conserved residues are shown in black, while lighter grays and white indicate various degrees of low conservation and no conservation, respectively. Conserved residues shared with other short-chain flavin reductases are marked with an asterisk at the bottom of the column. Color legend for sequence identity: green, conserved; yellow, similar; red, unconserved. Details of the proteins aligned with 3nfw are shown in Table 3 ▶.

A sequence alignment of *Mth*NTA-MoB with other members of the short-chain flavin reductase family is shown in Fig. 1 ▶. Sequences were selected either based on structural homology or owing to well reported functional similarity (Valton *et al.*, 2008 ▶; Filisetti *et al.*, 2003 ▶). *Mth*NTA-MoB shares many conserved residues within this family of short-chain flavin reductases, including Arg12, Gly35, Pro48, Leu76, Phe88, His130 and Leu152 (Fig. 1 ▶). However, the overall sequence similarity is low, despite high *E* values in structural classification. Table 3 ▶ lists the amino-acid identities and structural similarities of *Mth*NTA-MoB to a variety of other enzymes involved in the degradation of small organic compounds based on search results using *DALI* (Holm & Sander, 1993 ▶). In particular, the *Mth*NTA-MoB structure bears marked homology to the phenol 2-hydroxylase component B of reduced flavin reductase from *B. thermoglucosidasius* (*Bt*PheA2; PDB entry 1rz1; van den Heuvel *et al.*, 2004 ▶).

### 3.3. Three-dimensional structure of *Mth*NTA-MoB

**Figure 2:**
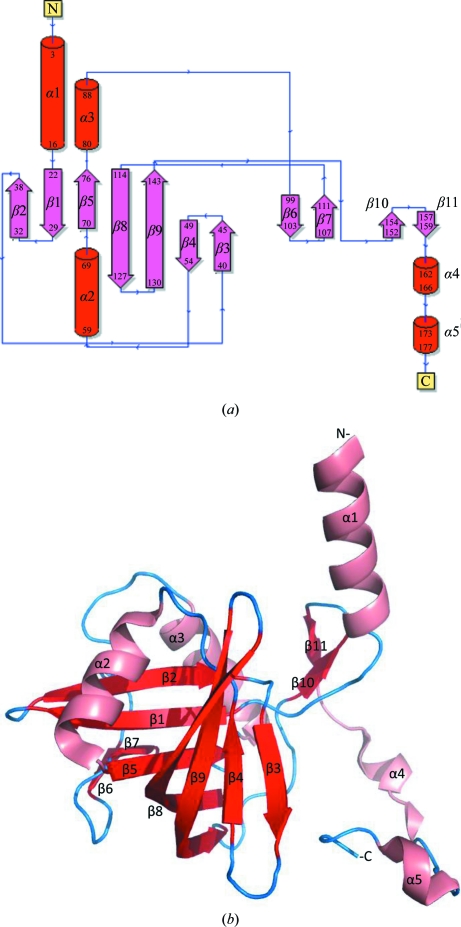
Structural diagrams of 3nfw. (*a*) Secondary-structural topology diagram. Red cylinders indicate α-helices and pink arrows represent β-sheets. The beginning and ending amino-acid residue numbers are indicated inside each of the cylinders or arrows, together with the sequential numbers of each secondary-structural component. (*b*) Ribbon diagram of 3nfw. Pink spirals indicate α-helices and red arrows represent β-sheets. Secondary-structural elements and N- and C-terminal residues are indicated.

The native *Mth*NTA-MoB protein crystallized in the orthorhombic space group *P*2_1_2_1_2_1_ and an apo structure was determined to a resolution limit of 1.6 Å (PDB entry 3nfw) with *R*_cryst_ and *R*_free_ factors of 0.186 and 0.217, respectively (Table 2 ▶). *Mth*NTA-MoB consists of 11 β-strands and five α-helices, with seven antiparallel β-sheet core regions forming a split-barrel motif capped by an α-helix (Fig. 2 ▶). Consistent with observations from size-exclusion chromatography, the biological molecule observed within the crystal lattice is a dimer and the crystal structure contained two noncrystallographically related dimers. *PISA* (Krissinel & Henrick, 2007 ▶) calculated a total buried surface area of 8610 Å^2^ and a change in solvent free energy of −234 kJ mol^−1^ upon dimerization.

**Figure 3:**
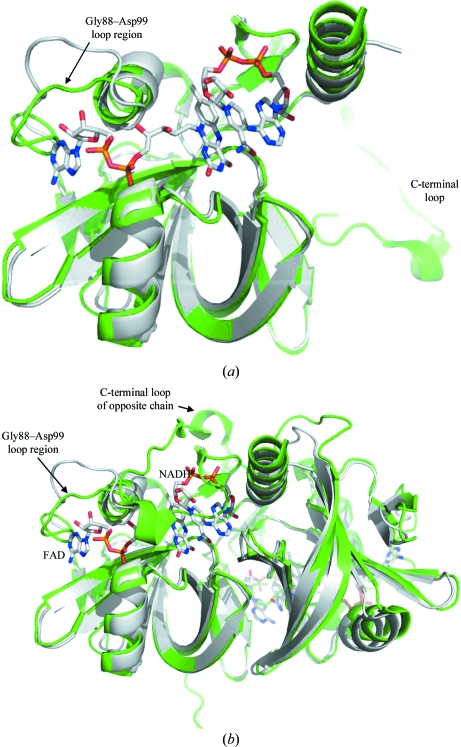
C^α^ trace of molecular superposition of 3nfw (green) and 1rz1 (gray). FAD and NADH molecules are shown bound to 1rz1. (*a*) Molecular overlay of the monomers, with the Gly88–Asp99 loop region and the C-terminal loop region indicated. (*b*) Molecular overlay of the dimers, with the Gly88–Asp99 loop region and the C-terminal loop region of the opposite chain and the FAD and NADH molecules bound to 1rz1 indicated.

The spatial difference between C^α^ atoms of *Mth*NTA-MoB and *Bt*PheA2 is 0.98 Å, indicating a high degree of structural conservation for two proteins with only 32% sequence similarity (Fig. 3 ▶). Unlike the *Mth*NTA-MoB structure, crystal data for *Bt*PheA2 were acquired with the cofactor NADPH bound. According to structure-alignment results generated using the *SSM* (PDBe) server (http://www.ebi.ac.uk/msd-srv/ssm/cgi-bin/ssmserver), the loop region at amino-acid residues Gly88–Asp99 (between α-helix 3 and β-strand 6) has the lowest level of amino-acid residue alignment. This loop forms a critical part of the binding pocket recognized by FMN, with the analogous loop reported to be involved in binding either FAD or FMN in PheA2 and ferric reductase, respectively (van den Heuvel *et al.*, 2004 ▶; Okai *et al.*, 2006 ▶). This loop has been described as flexible in the absence of FMN but highly ordered in FMN-bound structures. In the *Mth*NTA-MoB structure the loop region has the highest deviation from the structure of substrate-bound *Bt*PheA2. While the average distance between superimposed pairs of residues ranges from 1.29 to 4.42 Å, with the greatest deviation at Ala96, the overall C^α^ r.m.s.d. is only 0.98 Å (Fig. 3 ▶ *a*).

The other region in which *Mth*NTA-MoB deviates most from the *Bt*PheA2 structure is in the C-terminal loop region from Lys165 to Thr182 (Fig. 3*b*). This region adopts the same conformation in all four protomers in the asymmetric unit and is flexible and adjacent to the NADH-binding cleft (Deng *et al.*, 1999 ▶). It is expected to play an inhibitory role in substrate (flavin) and cofactor (NADH) binding (van den Heuvel *et al.*, 2004 ▶). However, further structural data are necessary to confirm the interaction of the loop with cofactor bound to the same molecule or a neighboring molecule.

## 4. Discussion

The reaction mechanism of this class of enzymes has been a topic of discussion in recent years (Filisetti *et al.*, 2003 ▶; van den Heuvel *et al.*, 2004 ▶; Valton *et al.*, 2008 ▶). One group proposed the ping-pong mechanism, while others described the reactions as following an ordered sequential mechanism. The flexible C-terminal region in the 3nfw structure could potentially play a role in inhibiting cofactor binding and/or release. However, without further evidence of a bound substrate or cofactor and a lack of functional analysis it is difficult to support either of the mechanisms one way or the nother.

Previous publications have suggested that lipid metabolism is fundamental to the unusual ability of *M. tuberculosis* to use fatty acids as a sole carbon source and survive *in vivo* (Van der Geize *et al.*, 2007 ▶). Recent reports further predict that *M. tuberculosis* subsists on alternative carbon sources in the human host. Specifically, *M. tuberculosis* is able to persist in the glucose-deficient and fatty-acid-rich environment found in the macrophage stage of infection, thereby contributing to its unparalleled virulence (Savvi *et al.*, 2008 ▶). The up-­regulation of the Rv3567c group of enzymes during survival in macrophages indicated their involvement in cholesterol uptake, which links them to phagocytosis, a vital step in bacterial pathogenic metabolism (Van der Geize *et al.*, 2007 ▶). An important role in cholesterol metabolism and cell survival could be the reason for the high conservation of these genes. Uncertainties remained in a previous discussion of the natural substrates of NTA-Mo (Uetz *et al.*, 1992 ▶). It is possible that the structure of the synthetic NTA and its subsequent degradation products resemble some of the key metabolites in metabolism pathways such as the tricarboxylic acid (TCA) pathway, hence the analogous activity. Further experiments to compare the binding affinities of these compounds and their reaction mechanism may help to answer some of these questions.

## 5. Conclusion

*Mth*NTA-MoB is 82% identical (89% similar) to *Mtu*NTA-MoB, which matches 100% to Rv3567c in TubercuList. Rv3567c and related cluster of genes, some of which are essential, have been assigned to the cholesterol-degradation pathway in *M. tuberculosis*. We have reported the 1.6 Å resolution structure of *Mth*NTA-MoB, one of the first protein structures to be reported for this organism. Structure comparisons characterize it as a member of the short-chain flavin reductase family with a high confidence level. The overall structure and conserved amino-acid residues align well with those of other proteins, despite low sequence homology. Two of the loop regions showed deviations in a structural overlay with other members of the family, including those in which the cofactor is bound. The density was sufficient to allow modelling of the long loop at the C-terminus, a region that is often absent in structures obtained using crystallo­graphic data owing to a high degree of flexibility, and may provide additional insight into its role in modulating activity *via* access for cofactor binding.

## Supplementary Material

PDB reference: NTA-MoB, 3nfw

## Figures and Tables

**Table 1:** Data-collection statistics Values in parentheses are for the highest of 20 resolution shells.

Space group	*P*2_1_2_1_2_1_
Unit-cell parameters (Å)	*a* = 48.5, *b* = 91.0, *c* = 165.6
Wavelength (Å)	1.000
Resolution range (Å)	50–1.6 (1.64–1.60)
No. of unique reflections	97350 (7102)
Multiplicity	8.2 (7.8)
Completeness (%)	99.8 (99.9)
*R*_merge_†	0.095 (0.428)
Mean *I*/σ(*I*)	13.4 (5.4)

† *R*_merge_ = 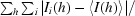 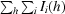.

**Table 2:** Refinement and model statistics Values in parentheses are for the highest of 20 resolution shells.

Resolution range (Å)	20–1.6 (1.64–1.60)
*R*_cryst_†	0.186 (0.180)
*R*_free_†	0.217 (0.227)
R.m.s.d. bonds (Å)	0.015
R.m.s.d. angles (°)	1.273
Protein atoms	6109
Heteroatoms	6
Waters	548
Mean *B* factor (Å^2^)	16.7
Residues in favored region (%)	97.6
Residues in allowed region (%)	100
*MolProbity*‡ score [percentile]	0.91 [100th]

† *R*_cryst_ = 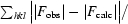 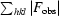. The free *R* factor was calculated with the 5% of the reflections that were omitted from the refinement (Winn *et al.*, 2011 ▶).

‡ Chen *et al.* (2010 ▶), Davis *et al.* (2007 ▶).

**Table 3:** Amino-acid identity and structural similarity across the top eight structural homologs of 3nfw available in the PDB Data based on the RCSB PDB (http://www.pdb.org/pdb/).

PDB code	UniProt ID	Protein name	Organism	Amino-acid identity (%)	R.m.s.d. (Å)	Reference
2rox	Q0I3S1	Putative flavin reductase	*Haemophilus somnus* 129PT	21.5	1.27	—
1rz1	Q9LAG2	Phenol 2-hydroxylase component B	*Bacillus thermoglucosidasius* A7	31	1.62	van den Heuvel *et al.* (2004 ▶)
2d37	Q974C9	Phenol 2-hydroxylase component B	*Sulfolobus tokodaii* strain 7	26	1.65	Okai *et al.* (2006 ▶)
2ecu	Q5SJP7	Flavin reductase (HpaC) of 4-hydroxyphenylacetate 3-monooxygenase polypeptide (L)	*Thermus thermophilus* HB8	27	1.73	Kim *et al.* (2008 ▶)
3cb0	Q8YHT7	4-Hydroxyphenylacetate 3-monooxygenase	*Brucella melitensis*	25.2	1.83	Lawrence *et al.* (2008 ▶)
1usc	P83818	Styrene monooxygenase small component	*Thermus thermophilus*	20	1.87	—
3k86	087008	Chlorophenol-4-monooxygenase component 2	*Burkholderia cepacia*	26	2.05	Webb *et al.* (2010 ▶)
2qck	A0JVA7	Flavin reductase domain protein	*Arthrobacter* sp. FB24	26	2.13	—
n/a	Q02058	Actinorhodin polyketide dimerase	*Streptomyces coelicolor* A3	32	n/a	Filisetti *et al.* (2003 ▶), Valton *et al.* (2008 ▶)
